# Expression of Concern: Hydroxychavicol, a *Piper betle* leaf component, induces apoptosis of CML cells through mitochondrial reactive oxygen species‐dependent JNK and endothelial nitric oxide synthase activation and overrides imatinib resistance

**DOI:** 10.1111/cas.16155

**Published:** 2024-03-27

**Expression of Concern:** Chakraborty, J.B., Mahato, S.K., Joshi, K., Shinde, V., Rakshit, S., Biswas, N., Choudhury (Mukherjee), I., Mandal, L., Ganguly, D., Chowdhury, A.A., Chaudhuri, J., Paul, K., Pal, B.C., Vinayagam, J., Pal, C., Manna, A., Jaisankar, P., Chaudhuri, U., Konar, A., Roy, S. and Bandyopadhyay, S. (2012), Hydroxychavicol, a *Piper betle* leaf component, induces apoptosis of CML cells through mitochondrial reactive oxygen species‐dependent JNK and endothelial nitric oxide synthase activation and overrides imatinib resistance. Cancer Science, 103: 88–99. https://doi.org/10.1111/j.1349‐7006.2011.02107.x

This Expression of Concern is for the above article, published online on 22 September 2011 in Wiley Online Library (wileyonlinelibrary.com), and has been published by agreement between the journal's Editor‐in‐Chief, Masanori Hatakeyama, the Japanese Cancer Association, and John Wiley and Sons Australia, Ltd. The Expression of Concern has been agreed due to concerns raised by a third party regarding duplicated panels (AG, SMTC) in Figure 4a and missing SD values in Figure 6 and Supplementary Figure 6. The authors admitted to the compilation error in Figure 4a and stated that they have repeated the Annexin V/PI assay with AG or SMTC alone or in combination with HCH using a similar protocol, which confirmed that AG or SMTC alone has no toxicity on K562 cells and these two inhibitors did not reverse the HCH mediated apoptosis of K562 cells. The authors also acknowledged their error of omitting the SD values in Figure 6 and Supplementary Figure 6. Subsequently, additional concerns have been raised about Figure 1e DAPI panels, that show high similarity with images from the authors’ previous publication, and about Figure 3b HCH subpanel, which includes highly similar cellular sections. The authors were unable to provide a satisfactory explanation, and due to the elapsed time, the original unmodified images and the raw data of this manuscript were no longer available. Therefore, the journal team could not verify the authenticity of the data and unambiguously exclude that these concerns affect the conclusions of the article. The journal has decided to issue an Expression of Concern to inform and alert the readers.

[Correction added on 14 September 2024, after first online publication: the Expression of Concern has been updated to include further concerns raised by a third party. More information can be found at https://doi.org/10.1111/cas.16357]

