# Correction to “Expression of concern: Hydroxychavicol, a *Piper betle* leaf component, induces apoptosis of CML cells through mitochondrial reactive oxygen species‐dependent JNK and endothelial nitric oxide synthase activation and overrides imatinib resistance”

**DOI:** 10.1111/cas.16357

**Published:** 2024-09-23

*Cancer Science* 115, no. 6 (2024): 2086–2086, https://doi.org/10.1111/cas.16155.

The originally published version of this Expression of Concern has been updated to include new information raised to us by a third party. The additional text is found below:

“Subsequently, additional concerns have been raised about Figure 1e DAPI panels, that show high similarity with images from the authors' previous publication, and about Figure 3b HCH subpanel, which includes highly similar cellular sections. The authors were unable to provide a satisfactory explanation, and due to the elapsed time, the original images and the raw data of the manuscript were no longer available.”

The online version of the originally published Expression of Concern has been corrected accordingly.

